# ‘*Candidatus* Mycoplasma Haemoalbiventris’ and Tick-Borne Pathogens in Black-Eared Opossum (*Didelphis aurita*) from Southeastern Brazil

**DOI:** 10.3390/microorganisms10101955

**Published:** 2022-09-30

**Authors:** Andrés Maurício Ortega Orozco, Lucas Drumond Bento, Pollyanna Cordeiro Souto, Fabricia Modolo Girardi, Bárbara Cristina Félix Nogueira, Ricardo Seiti Yamatogi, Artur Kanadani Campos, Carolyn Cray, Fabiano Montiani-Ferreira, Flávia Carolina Meira Collere, Thállitha Samih Wischral Jayme Vieira, Rafael Felipe da Costa Vieira, Leandro Abreu da Fonseca

**Affiliations:** 1 Veterinary Departament, Universidade Federal de Viçosa, Viçosa 36570-900, Brazil; 2 Division of Comparative Pathology, University of Miami Miller School of Medicine, Miami, FL 33136, USA; 3 Department of Veterinary Medicine, Universidade Federal do Paraná, Curitiba 80035-050, Brazil; 4 Vector-Borne Diseases Laboratory, Department of Veterinary Medicine, Universidade Federal do Paraná, Curitiba 80035-050, Brazil; 5 Global One Health initiative (GOHi), The Ohio State University, Columbus, OH 43210, USA

**Keywords:** hemoplasmas, *Hepatozoon* sp., Marsupial, Hematology, Biochemistry, ectoparasites

## Abstract

The black-eared opossum (*Didelphis aurita*) is a South American synanthropic marsupial. The presence of opossums in domestic spaces is relevant in the One-Health context since they are hosts of pathogens and ectoparasites that may affect the health of domestic animals and humans. In this study, we aim to determine the occurrence of hemoplasmas and selected tick-borne pathogens in free-ranging black-eared opossums, along with their molecular characterization, hematological and biochemical evaluation and factors associated with infection, in the municipality of Viçosa, State of Minas Gerais, southeastern Brazil. Thirty black-eared opossums were trapped between March 2021 and June 2022. Ectoparasites were collected. Hematological and biochemical analyses were performed. DNA from EDTA-blood samples were analyzed by PCR and qPCR assays. By molecular analyses, ‘*Candidatus* Mycoplasma haemoalbiventris’ was the most prevalent hemoparasite (73.3%), followed by *Hepatozoon* sp. (22.2%). Significant differences were observed in the number of platelets, and in the concentration of protein and globulins in the animals infected by ‘*Ca.* M. haemoalbiventris’ when compared with the negative group. This is the first report of ‘*Ca*. M. haemoalbiventris’ infection in *D. aurita*.

## 1. Introduction

The black-eared opossum (*Didelphis aurita*) is a synanthropic marsupial belonging to the genus *Didelphis* (Linnaeus, 1758) that inhabits the eastern region of Brazil, and is found mainly in the Atlantic Forest. Its distribution occurs as far as southeastern Paraguay and northeastern Argentina [1,2]. The presence of these marsupials in domestic spaces is relevant to the One-Health context since they are hosts of pathogens as bacteria, virus, protozoan and helminths that affect the health of domestic and wild animals and humans [3,4,5]. Data on hemotropic mycoplasmas (hemoplasmas) and tick-borne pathogens in opossums (*Didelphis* sp.) is scarce. Two species of hemoplasmas are known to infect *Didelphis*, ‘*Candidatus* Mycoplasma haemodidelphis’, detected in the Virginia opossum (*Didelphis virginiana*) from USA [6], and ‘*Candidatus* Mycoplasma haemoalbiventris’, detected in white-eared opossum (*Didelphis albiventris*) from Brazil [7,8,9,10]. 

In the Americas, different tick-borne pathogens (TBP) have been described to infect *Didelphis* sp. In 1973, Ayala described *Hepatozoon didelphidys* in the common opossum (*Didelphis marsupialis*) in Colombia [11] and De Thoisy et al. [12] visualized *Hepatozoon* sp. gamonts in the blood smear of *D. marsupialis* and *D. albiventris* in the French Guyana. In the United States, *Hepatozoon* sp. has been molecularly detected in the blood of *D. virginiana* [13]. In Brazil, Da Silva et al. [14] and Colle et al. [15] also identified *Hepatozoon* sp. in *D. albiventris* and *D. marsupialis*, respectively. *Babesia* sp. have been found in *D. albiventris* [16] and in *D. marsupialis* from Brazil [15,17]. Additionally, novel ehrlichial agents have been described in *D. albiventris*, *Ehrlichia* sp. strain Natal in the Brazilian northeastern region [18] and *Ehrlichia* sp. in Campo Grande, Mato Grosso do Sul State [19], central-western Brazil. 

In Brazil, different tick species have been reported to parasitize *D. albiventris* and *D. aurita* such as *Amblyomma sculptum*, *Amblyomma dubitatum*, and *Ixodes loricatus* [20,21,22]. Although different TBP have been described infecting *Didelphis* sp. [5,13,14,15,16,19,23,24], information on hemoplasmas and TBP on *D. aurita* still are scarce. In addition, the impact that these microorganisms may have on the health of affected individuals as well as the possible changes on hematological and biochemical profile remain unknown. Therefore, the aims of this study were (i) to determine the occurrence of hemoplasmas and selected TBP, (ii) to identify tick species parasitizing animals, and (iii) to evaluate the hematological and biochemical profile in free-ranging *D. aurita*, in the municipality of Viçosa, State of Minas Gerais, southeastern Brazil.

## 2. Material and Methods

### 2.1. Study Area

**Figure 1:**
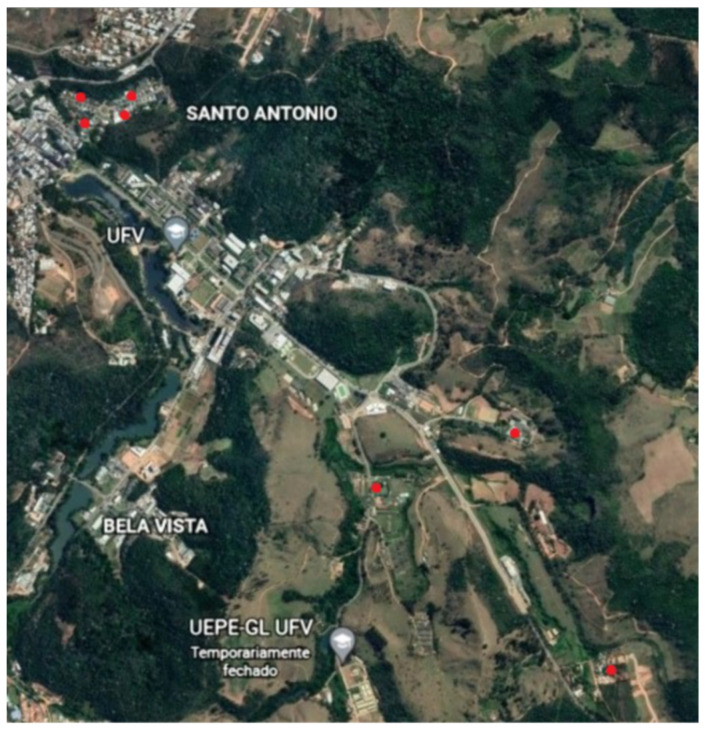
Black-eared opossum (*Didelphis aurita*) capture sites, Viçosa municipality, Minas Gerais State, southeastern Brazil.

The study was carried out in the Viçosa municipality (22°45′14″ S and 42°52′55″ W), Minas Gerais State, in the Southeast Region of Brazil (Figure 1). The municipality has an area of 299,418 km^2^ and a has a humid subtropical climate, category Cwa by Kӧppen and Geiger, with an average temperature of 20.4 °C and average rainfall of 1251 mm/year [25].

### 2.2. Sampling

A total of 30 *D. aurita* were captured using Tomahawk traps baited with sardines, cornmeal, and bananas. Sampling was performed between March 2021 and June 2022. The animals were physically restrained for clinical examination [26] and blood sampling. Blood samples (up to 2 mL) were collected by venipuncture of the coccygeal lateral vein [27] using sterile EDTA-coated tubes (Becton & Dickinson Co, Franklin Lakes, NJ, USA) for hematological analyses, and later kept at −20 °C until PCR testing. Additionally, two milliliters were placed into tubes without anticoagulant (Becton & Dickinson Co, Franklin Lakes, NJ, USA) and kept at room temperature (25 °C) until visible clot formation. The samples were then centrifuged at 1500 × *g* for 5 min, serum separated and kept at −20 °C for biochemical analyses.

The animals also were submitted to a visual inspection for presence of ectoparasites (ticks and fleas). Ticks were removed using a commercial Hook and kept in 70% ethanol labeled tube for identification according to previously described morphological taxonomic keys [28].

### 2.3. Hematological and Biochemical Analyses

For complete blood cell count (CBC) the samples were analyzed with an automatic cell counter (URIT 3000^®^, MHLab^®^, Sao Paulo, Brazil). The packed cell volume (PCV) was determined by the micro-hematocrit technique, with animals considered anemic when <0.31 L/L [29]. Total plasma protein (TPP) concentration was determined by refractometer. For leukocyte evaluation, blood smears were stained using a Romanowsky-type stain (Panotico: Instant Prov (Newprov^®^, Pinhais, Paraná, Brazil) and examined using light microscopy (CX40, Olympus, Tokyo, Japan) adapted to a camera connected to the ToupView software (ToupTek, Zhejiang, China) under 1000× magnification. CBC data were expressed through the mean and standard deviation (SD). 

The serum biochemical parameters evaluated were albumin (ALB; bromocresol green), total protein (TP; biuret method), Alanine Aminotransferase (ALT; method: UV kinetic), Aspartate Aminotransferase (AST; method: UV kinetic), Phosphatase Alkaline (PA; Method: optimized kinetic), Gamma glutamyl transferase (GGT; kinetic method), urea (method: kinetic) and creatinine (method: alkaline picrate) obtained through spectrophotometry in an automatic biochemical analyzer (HumaStar 200^®^, In Vitro Diagnóstica, Belo Horizonte, Brazil) with specific commercial reagents. The results obtained were expressed through the mean and SD.

### 2.4. Morphometry

The length, width and area of the structures and cells were measured using Image-Pro Plus 4.5 software (Media Cybernetcs1, Silver Spring, MD, USA).

### 2.5. DNA Extraction

DNA from 200 μL whole blood was extracted using a commercial kit (MagMAX^TM^ CORE Nucleic Acid Purification Kit, TX, USA), according to the manufacturer’s instructions. Ultrapure water was used in parallel as a negative control to monitor cross contamination. To monitor DNA extraction, a conventional PCR assay targeting a fragment of the mammalian endogenous gene glyceraldehyde-3-phosphate dehydrogenase (*gapdh*) [30] was performed in all samples.

### 2.6. PCR Assays

Black-eared opossums’ DNA samples were initially screened using a universal hemoplasma SYBR green real-time PCR (qPCR) assay, as previously described [31]. Samples with threshold cycle (C*_T_*) value < 32 were considered positive [32] and then submitted to conventional PCR assays targeting a fragment (900 bp) of the 16S rRNA [33,34] and (800 pb) of the 23S rRNA genes of hemoplasmas [35]. Nuclease-free water and *Mycoplasma ovis* DNA obtained from a naturally infected goat (*Capra hircus*) [35] were used as negative and positive controls, respectively.

Moreover, black-eared opossums’ DNA samples were also screened by conventional PCR assays targeting a fragment (667 bp) of the 18S rRNA gene of *Hepatozoon* spp. [26] and a fragment (349 bp) of the *dsb* gene of *Ehrlichia* spp. [36]. *Hepatozoon canis* and *Ehrlichia canis* DNA obtained from naturally infected dogs and nuclease-free water were used as positive and negative controls, respectively. 

### 2.7. Sequencing and Phylogenetic Analysis

Amplicons (∼900 bp) obtained from two hemotropic *Mycoplasma* sp.-positive samples were sequenced in both directions by Sanger method, with nucleotide sequences of the 16S rRNA submitted to GenBank^®^ database (accession numbers: OP279616, OP279617). The partial sequences of the 16S rRNA gene of hemotropic *Mycoplasma* sp. were aligned with other hemotropic *Mycoplasma* sp. species available in GenBank^®^ using MAFFT 7.110 [37] on the Guidance 2 server [38]. Phylogenetic analyses on the 16S rRNA gene was performed based on Bayesian inference using the Beast 1.8.0 package [39]. Three independent runs of 100,000,000 generations of Monte Carlo Markov Chain (MCMC) were performed, with one sampling per 10,000 generations and a 10% burn in. The substitution model was estimated as GTR + I + G based on the Akaike information criterion (AIC) using jModeltest 2.1.10 [40]. Reconstructions were visualized using the FigTree 1.4.4 software [41]. The 16S rRNA gene tree was rooted with *Mycoplasma pneumoniae* (CP039761). Moreover, a haplotype network for each fragment was estimated in the PopArt 1.7 [42] using the median-joining method [43].

Amplicons (740 bp) obtained from five *Hepatozoon* sp.-positive samples were sequenced in both directions by Sanger method, with nucleotide sequences of the 18S rRNA submitted to GenBank^®^ database (accession numbers: OP267564–OP267568). The partial sequences of the 18S rRNA gene of *Hepatozoon* sp. were aligned with other *Hepatozoon* species available in GenBank^®^ using MAFFT 7.110 [37] on the Guidance 2 server [38]. Phylogenetic analyses on the 18S rRNA gene was performed based on Bayesian inference using the Beast 1.8.0 package [39]. Three independent runs of 100,000,000 generations of Monte Carlo Markov Chain (MCMC) were performed, with one sampling per 10,000 generations and a 10% burn in. The substitution model was estimated as GTR + G based on the AIC using jModeltest 2.1.10 [40]. Reconstructions were visualized using the FigTree 1.4.4 software [41]. The 18S rRNA gene tree was rooted with *Plasmodium vivax* (XR_003001206). Moreover, a haplotype network for each fragment was estimated in the PopArt 1.7 [42] using the median-joining method [43].

### 2.8. Statistical Analysis

To compare the means, t-test (red blood cells, hemoglobin, PCV, MCH, MCHC, RDW, platelets, ALB, TP, globulins, albumin:globulin ratio, creatinine, glucose) was used. Data normality was verified by the Shapiro-Wilk test. Non-parametric analyzes for non-normal variables (MVC, white blood cells, neutrophils, lymphocytes, monocytes, band cells, basophils, length, width, area, TP, fibrinogen, PA, ALT, AST, urea, gamma-glutamyl transferase) were analyzed using the Mann-Whitney test. A significance level of 5% was considered for all analyses. A Fisher’s exact test was used to determine whether individual variables (gender, age group and presence of ectoparasites) was associated with the infection. Odds ratio (OR), 95% confidence interval and *p*-values were calculated for each variable. Results were considered significantly different when *p* < 0.05. Data were compiled and analyzed in the software GraphPad Prism (version 9.0, Graphpad, San Diego, CA, USA).

### 2.9. Ethical Aspects 

The study was approved by the Ethics Committee for the Use of Animals-CEUA/UFV by process number 30/2021 under the regulations of the Chico Mendes Institute for Biodiversity Conservation (ICMBio, license number 64930-3).

## 3. Results

**Figure 2:**
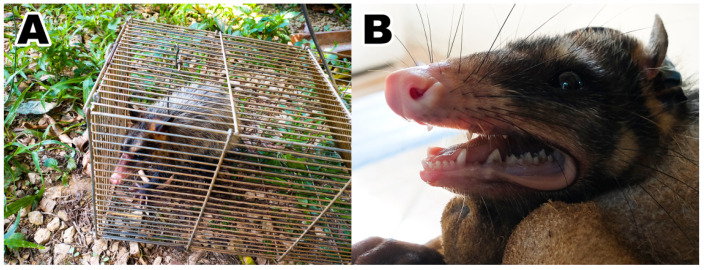
A black-eared opossum (*Didelphis aurita*), male, adult. (**A**) Inside the Tomahawk trap. (**B**) During physical restraint.

Out of the 30 animals captured, 15 (50.0%) were adult females, four (13.3%) sub-adult females, eight (26.6%) adult males and three (10.0%) sub-adult males (Figure 2). A total of 14/30 (46.66%; 95% CI = 30.23–63.86) black-eared opossums were infested by ticks at the time of sampling. Ticks were identified as *I. loricatus* (seven F and three M), *Amblyomma ovale* (three nymphs), *A. dubitatum* (one nymph) and *Amblyomma* sp. (21 larvae).

### 3.1. Hematological and Biochemical Analyses

The mean and range values obtained in the CBC and biochemical analyses of black-eared opossums are summarized in Table 1. A total of three/30 (10.0%; 95% CI = 3.46–25.62) black-eared opossums were anemic. 

**Figure 3:**
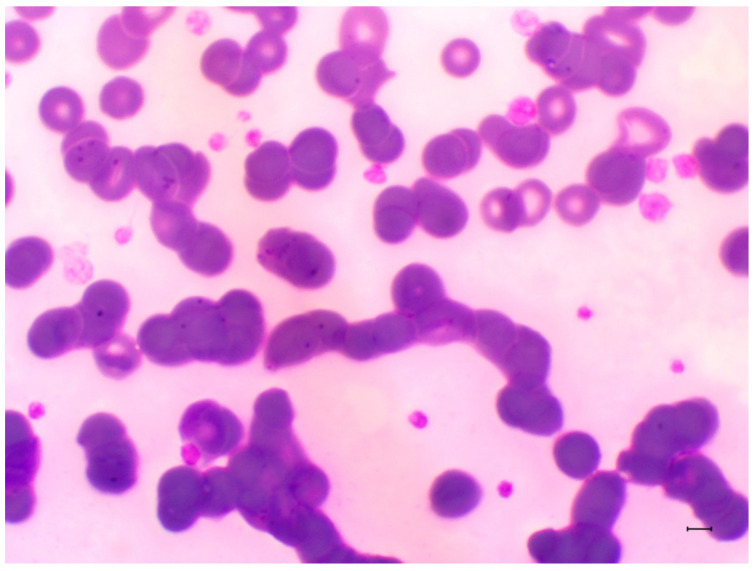
Light microscopy image of Romanowsky-type-stained peripheral blood smear from black-eared opossum (*Didelphis aurita*), showing small basophilic and round structures attached to erythrocytes (arrows). Sample from adult female black-eared opossum (1000× magnification). Bar = 10 µm.

**Figure 4:**
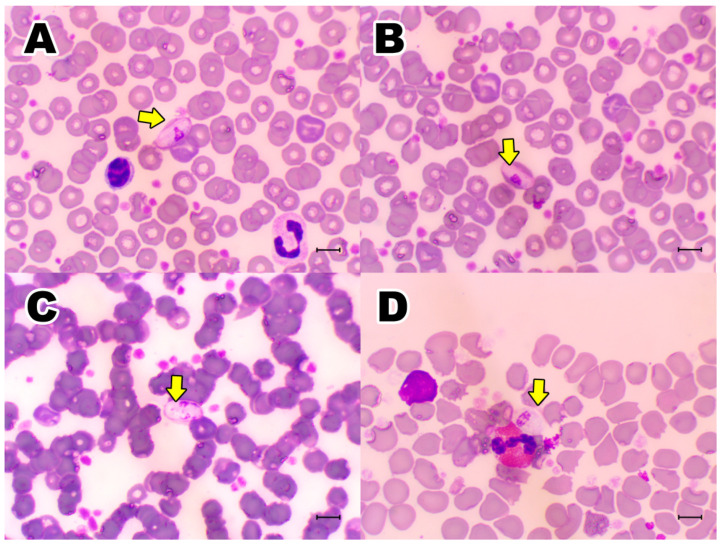
Light microscopy image of Romanowsky-type-stained peripheral blood smear from black-eared opossum (*Didelphis aurita*), showing (**A**,**B**) intra-erythrocyte *Hepatozoon* sp. gamonts (arrow), and (**C**,**D**) free (between cells). Sample from female adult (**A**,**B**,**D**) and a male adult (**C**) black-eared opossum (1000× magnification). Bar = 10 µm.

During blood smears evaluation, hemoplasma-like structures were visualized on the surface of red blood cells (RBCs) from one/30 (3.33%; 95% CI = 0.05–16.67) black-eared opossums (Figure 3). Additionally, 15/30 (50.0%; 95% CI = 33.15–66.85) animals showed *Hepatozoon* sp. gamonts on blood smears evaluation (Figure 4). 

*Hepatozoon* sp. gamonts found on blood smears presented variations: they were mainly oval, but it was also possible to identify some with a more rounded shape and others with a banana-like shape (Figure 4A–D). The mean and SD measures of *Hepatozoon* sp. gamonts were length: 10.89 ± 0.92 µm; width: 5.29 ± 0.97 µm; area: 48.51 ± 7.72 µm. When present, the pleomorphic nucleus mean measures were length: 3.90 ± 1.05 µm and width: 2.03 ± 0.81 µm. Measurements were performed on *Hepatozoon* sp. gamonts inside RBCs and those free on blood smears. When RBCs were infected with *Hepatozoon* sp. gamonts, cells showed the following mean and SD measures length: 11.7 ± 0.74; Width: 6.83 ± 1.19 µm; Area: 64.53 ± 7.93 µm. RBCs that were not infected presented the following mean and SD measures length: 7.88 ± 0.71; Width: 7.40 ± 0.64 µm; Area of 45.74 ± 7.63 µm. Measures of RBCs infected with *Hepatozoon* sp. gamonts differed significantly from non-infected RBCs in the length (*p* < 0.0001), width (*p* < 0.0001) and area (*p* < 0.0001).

### 3.2. PCR Assays

**Figure 5:**
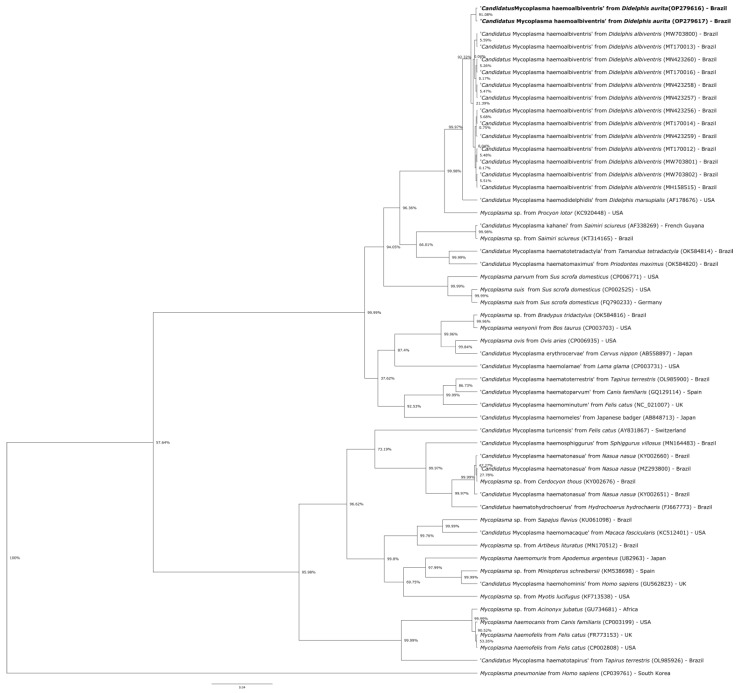
Phylogenetic tree based on partial sequences of the 16S rRNA gene (850 bp), showing the relationship between the hemotropic *Mycoplasma* sp. detected in the black-eared opossums (*Didelphis aurita*) from this study and other hemoplasmas. Mycoplasma pneumoniae was used as outgroup. The GenBank accession number is in parentheses after the species name and origin of each agent. Bayesian inferences were carried out applying the GTR + I + G model and 1000 bootstrap replicates for all analyses.

The *gapdh* gene was consistently amplified in all black-eared opossum samples. A total of 22/30 (73.3%; 95% CI = 55.55–85.82) animals were positive for hemotropic *Mycoplasma* sp. by the qPCR. The animal that hemoplasma-like structures were visualized on blood smear evaluation has also tested positive by qPCR. Sequencing of the 16S rRNA gene fragments from two hemoplasma-positive black-eared opossums revealed 98.87–98.96% identity with ‘*Ca.* M. haemodidelphis’ detected in *D. albiventris* from Brazil (AF178676). Phylogenetic analyses on the 16S rRNA gene of the hemoplasma detected herein clustered together with those of the *Mycoplasma suis* group. Hemoplasma sequences obtained from *D. aurita* clustered together with ‘*Ca*. M. haemoalbiventris’ sequences previously detected in *D. albiventris* from southern Brazil (Figure 5). 

**Figure 6:**
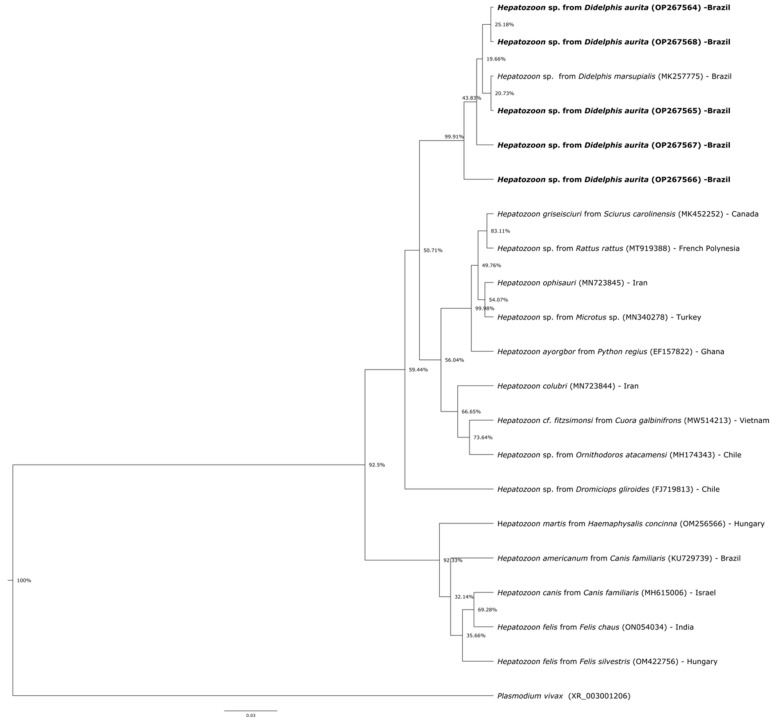
Phylogenetic tree based on partial sequences of the 18S rRNA gene (740 bp), showing the relationship between the *Hepatozoon* sp. detected in the black-eared opossums (*Didelphis aurita*) from this study and other *Hepatozoon* species. Mycoplasma pneumoniae was used as outgroup. The GenBank accession number is in parentheses after the species name and origin of each agent. Bayesian inferences were carried out applying the GTR + I + G model and 1000 bootstrap replicates for all analyses.

Twelve out of 30 (40%; 95% CI = 24.59–57.68) *D. aurita* tested positive for *Hepatozoon* sp. by PCR. Seven out of 15 (46.67%; 95% CI = 24.81–69.88) animals that showed *Hepatozoon* sp. gamonts on blood smears evaluation also tested positive for *Hepatozoon* sp. by PCR. A total of eight/30 (26.67% 95% CI = 14.18–44.45) tested positive for hemoplasmas and *Hepatozoon* sp. by molecular assays, however animals did not show any clinical signs of infection. Sequencing of the 18S rRNA gene fragments from five *Hepatozoon*-positive *D. aurita* showed 96.2–96.4% identity with *Hepatozoon colubri* detected in the aesculapian snake (*Zamenis lineatus*) from Iran (MN723844.1). Phylogenetic analysis inferred by Bayesian inference and based on a 740 bp alignment (Figure 6) demonstrate the close relationship of the *D. aurita Hepatozoon* sp. with *Hepatozoon* sp. genotypes detected in *D. marsupialis* from Brazil (GenBank^®^ accession no. MK257775). 

Two out of 30 (6.66%; 95% CI = 1.85–21.32) animals tested positive for *Ehrlichia* sp. by PCR. Sequencing of the *dsb* gene fragment (165 bp) from one *Ehrlichia*-positive *D. aurita* showed 99.38% identity with *Ehrlichia chaffeensis* detected in *Hyalomma excavatum* tick from Egypt (MN372100) and *Odocoileus virginianus* from USA (MK611628).

### 3.3. Statistical Analysis

In hemoplasma-positive *D. aurita*, the number of platelets were lower (*p* < 0.05) and the TPP concentration showed a significant increase (*p* < 0.05) (Table 2). In the biochemical analyses, a similar result was evidenced. The concentration of TP and globulins, as well activity of Alkaline Phosphatase (AP) showed significant increased (*p* < 0.05) in the hemoplasma-positive animals (Table 2).

In the animals infected by *Hepatozoon* sp. no significant differences were observed for hematological analyses and in the biochemical analyses, only creatinine showed a significant increase (*p* < 0.05) in the positive group. No significant associations (*p* > 0.05) were found between gender, age group or presence of ectoparasites and positivity to hemoplasmas or *Hepatozoon* sp. (Table 3). 

## 4. Discussion

To the best of authors knowledge, this is the first study on hemoplasmas in *D. aurita*. Previous studies have reported ‘*Ca.* M. haemoalbiventris’ in *D. albiventris* from different Brazilian regions and biomes with prevalence data ranging from 32.5–87.5% by PCR [7,8,9,10]. Herein, 73.3% *D. aurita* tested positive for ‘*Ca.* M. haemoalbiventris’ by qPCR. Additionally, ‘*Ca.* M. haemoalbiventris’ was observed by light microscopy of black-eared opossum’s blood smear as small basophilic epierythrocytic structures of coccoid shape, individually attached and often one per erythrocyte (Figure 3). 

The main laboratory alterations were observed in hemoplasma-positive animals. Messick et al. [6] stated that infection by *’Ca*. M. haemodidelphis’ produces severe anemia in *D. virginiana*. In the present study, this was not evident and the values of PCV, hemoglobin and RBCs were higher than those previously reported in *D. aurita* and *D. albiventris* [27], and lower than the values obtained by Lewis [44]. Herein, an association between hemoplasma-positive animals and a decrease in the number of platelets was observed. A previous study has described that one of the mechanisms involved in thrombocytopenia in cases of *Mycoplasma pneumoniae* infection would be related to the production of antibodies against platelets which would induce their destruction by the immune system [45]. In the present study an increased TPP concentration in hemoplasma-positive animals was observed (*p* < 0.05), that may be related to the antigenic stimulation of pathogens detected [46,47,48]. Whether ‘*Ca.* M. haemoalbiventris’ is capable of producing a clinical disease similar to *’Ca*. M. haemodidelphis’ in *D. virginiana* [6] remains to be fully established. 

In the biochemical analysis, in the hemoplasmas group, we had similar results as observed in the CBC. The concentration of TP (*p* < 0.05) and globulins (*p* < 0.05) were higher in animals infected with ‘*Ca.* M. haemoalbiventris’ than in the negative groups. The mean value of TP obtained in this study was higher at 5.6 ± 0.6 g/dL described by Carvalho Do Nascimento and Horta [27] and the 7.1 g/dL of Lewis [44]. The increase in TP concentration may be a consequence of hyperglobulinemia [46,47,48]. The main proteins present in serum are albumin and globulins. Albumin is a hepatic synthesis protein and among its functions are the transport of substances and the regulation of oncotic pressure. Globulins correspond to a diverse group of proteins such as immunoglobulins, complement system proteins, fibrinogen and others [47].

One of the main causes of hyperglobulinemia are inflammatory processes, either acute or chronic, which can result as a consequence of an increase in one or more globulins [46]. In this case, the infected opossums could have an increase in globulins by the antigenic stimulation that the hemoplasmas produce in the organism of the affected individuals.

The activity of AP was higher (*p* < 0.05) in the animals that were not infected with ‘*Ca.* M. haemoalbiventris. Interpreting AP results can be a challenge. The only available reference [27] showed mean values of 11.9 ± 4.2 U/L, much lower than ours. In the physical or laboratory evaluation, it was not possible to identify a cause of those commonly associated (hepatobiliary disease, osteoblastic activity or cortisol induction) to increases AP activity [47] that could justify the variations, therefore, perhaps it is a case of a statistical difference, but not a biological.

In the present study, 50% *D. aurita* presented *Hepatozoon* sp. gamonts by light microscopy of blood smears and 40% tested positive by the PCR targeting the 18S rRNA gene of *Hepatozoon* species. Sequencing confirmed that animals were infected by *Hepatozoon* sp. closely related to *Hepatozoon* sp. detected in *D. marsupialis* from Brazil [15]. Previous studies have reported different *Hepatozoon* species in marsupials [15,23,49,50] and *H. canis* in *D. albiventris* from São Paulo State, southeastern Brazil [14]. Additionally, putative new *Hepatozoon* sp. have been found in *D. marsupialis* [15] and *D. albiventris* from Brazil [19], with an overall prevalence of 45.16% (14/31) and 2.3% (1/43), respectively. Phylogenetic analysis showed that four *Hepatozoon* sp. sequences detected in *D. aurita* herein clustered apart from *Hepatozoon* sp. detected in *D. marsupialis* from Brazil. Future studies should be performed to elucidate and characterize *Hepatozoon* sp. infecting black-eared opossums from Brazil.

The morphometric analyzes of the *Hepatozoon* sp. gamonts detected herein were similar to those described for *H. didelphydis* [51] found as intra-erythrocytic and free parasites, with spherical or ovoid form with 8–10 × 4–6 µm in the blood from one male black-eared opossum (*n* = 50) in the city of São João de Meriti, Rio de Janeiro State, southeastern Brazil. In Colombia, in the Eastern Llanos region, Ayala et al. [11] described two types of Haemogregarines. In the first case, *D. marsupialis*, *Philander opossum* and *Metachirus nudicaudatus* showed *H. didelphydis* on stained blood smears with an average size of 10.5 × 5.0 µm in 10 animals, similar to our findings. In the second group, gamonts of an unidentified Haemogregarine were visualized in the blood smear of *D. marsupialis* at a different capture site with an average measurement of 11.7 × 6.0 µm (*n* = 20). According to the authors, the main difference was that in the second group, the RBCs were considerably hypertrophied, and in the first group they only showed a slight increase. In a similar way in the present study, the RBCs that were infected with gamonts were also hypertrophied when compared with not infected cells. 

De Thoisy et al. [12] also described two types of *Hepatozoon* sp. in mammals from French Guyana. In the first case, infecting erythrocytes of *D. marsupialis*, *P. opossum* and *M. nudicaudatus* which increased the red cells size, with a prevalence of 25%, 57% and 5% respectively and that could be related to *H. didelphydis*, perhaps the same species described by Ayala et al., [11]. In that study, *D. albiventris* had an infection prevalence of 17%. In this species, the gamonts had measurements of 11.2 ± 1.2 × 8.0 ± 1.1 μm and a pleomorphic nucleus of 7.7 ± 0.9 × 5.1 ± 0.4 μm and according to the authors, it could be a new species. In the study carried out by Da Silva et al. [14] was found in the blood smear of one animal a *Hepatozoon* gamont that presented 8.88 × 5.17 μm and the nucleus 3.84 × 3.25 μm. In the other hand, André et al. [19] did not identified any gamont in the blood smears of the *D. albiventris* evaluated. 

In the animals infected with *Hepatozoon* sp. there was no difference in the CBC parameters even when the degree of parasitemia varied among individuals, from a few to countless gamonts (Figure 4A–D). The only difference in the biochemical analysis in the *Hepatozoon* sp. group was observed in the creatinine value, which was higher in the positive group. The mean value of creatinine was practically identical to the 0.05 ± 0.6 mg/dL mentioned by Carvalho Do Nascimento e Horta [27] used as a reference. In the absence of significant differences in the CBC or biochemical analyzes, it is valid to consider the hypothesis that this *Hepatozoon* sp. is well adapted to the host because it does not produce an evident clinical disease. To better evaluate this hypothesis, an investigation with a greater number of opossums should be carried out. Perhaps the infection may be similar to dogs with *H. canis*, and in agreement with what was proposed by Baneth et al., [52] who suggest that this protozoan is well adapted to dogs that, in case of producing disease, it will be subclinical or mild. 

## 5. Conclusions 

A high occurrence of ‘*Ca*. M. haemoalbiventris’ and *Hepatozoon* sp. was found in *D. aurita* from the municipality of Viçosa, Minas Gerais State. Infection was not associated with sex, age group and/or the presence of ectoparasites. Infection by ‘Ca. M. haemoalbiventris’ was associated with lower number of platelets, an increase in TPP, TP and globulins concentration. Whether infection by these agents may cause clinical disease in *D. aurita* remains to be fully established.

## Figures and Tables

**Table 1:** Mean, standard deviation and range of complete blood count (CBC) and serum biochemical results of black-eared opossums (*Didelphis aurita*) (*n* = 30).

**Erythrocytes**		
**Analyte**	**Mean ± SD**	**Range (Min-Max)**
Red blood cells (×10^6^/µL)	4.31 ± 1.06	1.68–7.33
Hemoglobin (g/dL)	11.77 ± 1.99	5.80–15.60
PCV (%)	38.15 ± 6.47	19.00–50.30
Total protein (g/dL)	7.41 ± 0.85	5.80–9.60
MVC (fl)	84.00 ± 8.17	65.50–110.10
MCH (pg)	28.47 ± 5.28	16.23–39.82
MCHC (%)	30.91 ± 1.84	26.90–34.23
RDW (%)	10.02 ± 0.69	8.70–11.40
Fibrinogen (g/dL)	0.23 ± 0.09	0.20–0.60
**Leukocytes**		
**Analyte**	**Mean ± SD**	**Range (Min-Max)**
Leukocytes /µL	14,596.67 ± 6200.58	5800–36,700
Neutrophils /µL	6755.37 ± 3614.52	2300–17,616
Lymphocytes /µL	5306.87 ± 2499.46	456–11,233
Monocytes /µL	416.43 ± 493.10	73–2202
Band /µL	60.33 ± 158	0–734
Eosinophils /µL	1790.77 ± 1603.63	0–6606
Basophils /µL	202.30 ± 246.69	0–1088
**Platelets**		
**Analyte**	**Mean ± SD**	**Range (Min-Max)**
Platelets ×10^3^/µL	284.96 ± 173.23	32–716
**Serum biochemical analyses**		
**Analyte**	**Mean ± SD**	**Range (Min-Max)**
Albumin (g/dL)	3.02 ± 0.44	1.85–3.75
Total protein (g/dL)	7.58 ± 1.12	5.49–9.33
Globulins (g/dL)	4.56 ± 1.20	2.77–7.48
Albumin:globulin ratio	0.70 ± 0.20	0.24–1.09
AP (U/L)	1009.30 ± 855.14	223.00–3345.00
ALT (U/L)	65.82 ± 48.19	29.80–226.40
AST (U/L)	192.93 ± 162.55	3.00–994.00
Urea (mg/dL)	71.37 ± 17.44	50.00–292.00
Creatinine (mg/dL)	0.49 ± 0.19	0.00–0.76
GGT (U/L)	31.00 ± 11.58	18.00–65.00
Glucose (mg/dL)	120.71 ± 46.76	44.80–256.60

SD: standard deviation; PA: alkaline phosphatase; ALT: alanine aminotransferase; AST: aspartate aminotransferase; GGT: gamma glutamyl transferase.

**Table 2:** Significant differences * (*p* < 0.05) in the concentration of some of the analytes evaluated of *D. aurita* with hemoplasma infection.

Hematology			
Analyte	Negative	Positive	*p*-Value
Red blood cells (×10^6^/µL)	4.596 ± 1.259	4.199 ± 0.989	0.3734
Hemoglobin (g/dL)	11.65 ± 0.89	11.81 ± 2.28	0.8465
PCV (%)	38.51 ± 2.90	38.02 ± 7.41	0.8570
Total protein (g/dL)	6.800 ± 0.59	7.800 ± 0.79	0.0083 *
MVC (fl)	85.75 ± 7.8	84.40 ± 8.34	0.9542
MCH (pg)	27.53 ± 6.67	28.81± 4.81	0.5642
MCHC (%)	30.34 ± 2.51	31.12 ± 1.55	0.3111
RDW (%)	10.36 ± 0.64	9.900 ± 0.67	0.1034
Fibrinogen (g/dL)	0.2000 ± 0.07	0.2000 ± 0.09	>0.9999
Leukocytes /µL	13,250 ± 4724.69	13,000 ± 6751.73	0.9817
Neutrophils /µL	5636 ± 3729.82	6341± 3647.43	0.6622
Lymphocytes /µL	4655 ± 1857.24	5265 ± 2733.69	>0.9999
Monocytes /µL	187.0 ± 235.15	243.5 ± 551.48	0.3156
Band /µL	72.75 ± 148.51	55.81 ± 164.58	0.8620
Eosinophils /µL	1514 ± 1729.28	1320 ± 1598.27	0.9451
Basophils /µL	167.0 ± 227.87	165.5 ± 258.27	>0.9999
Platelets ×10^3^/µL	452,500 ± 184,945	224,045 ± 124,344	0.0006 *
**Serum biochemical analyses**			
Albumin (g/dL)	2.779 ± 0.15	3.005 ± 0.39	0.1271
Total protein (g/dL)	6.370 ± 0.56	7.559 ± 1.03	0.0048 *
Globulins (g/dL)	3.591 ± 0.60	4.555 ± 1.11	0.0285 *
Albumin:globulin ratio	0.7935 ± 0.13	0.7035 ± 0.20	0.2579
AP (U/L)	1932 ± 827.10	857.0 ± 706.91	0.0021 *
ALT (U/L)	46.90 ± 11.76	55.00 ± 48.09	0.1154
AST (U/L)	117.5 ± 152.61	233.5 ± 229.33	0.2371
Urea (mg/dL)	78.65 ± 19.19	73.50 ± 59.99	0.7214
Creatinine (mg/dL	0.4600 ± 0.15	0.4591 ± 0.19	0.9905
GGT (U/L)	27.50 ± 7.34	27.00 ± 12.46	0.6525
Glucose (mg/dL)	126.4 ± 51.13	126.4 ± 69.34	0.9994

**Table 3:** Prevalence of hemotropic mycoplasmas and *Hepatozoon* sp. in *D. aurita* within each variable studied, Viçosa, Minas Gerais, Brazil.

**Hemotropic Mycoplasmas**				
**Variable**	**+/*n***	**OR (95% CI)**		***p*-Value**
Gender				
Male	7/11 (63.33)	0.4667 (0.1066–2.036)		0.4172
Female	15/19 (78.94)			
Age group				
Adult	17/23 (73.91)	1.1333 (0.1858–5.956)		>0.9999
Sub adult	5/7 (71.42)			
Presence of ectoparasites				
Ectoparasite	17/21 (80.95)	1.7000 (0.2578–11,58)		0.6219
Without ectoparasite	5/9 (55.55)			
***Hepatozoon* sp.**				
**Variable**	**+/*n***		**OR (95% CI)**	***p*-Value **
Gender				
Male	3/10 (30.00)		2.0000 (0.3769–10.13)	0.6382
Female	3/17 (17.64)			
Age group				
Adult	5/20 (25.00)		2.0000 (0.2494–27.07)	>0.9999
Sub adult	1/7 (14.28)			
Presence of ectoparasites				
Ectoparasite	4/19 (21.05)		0.8000 (0.1267–5.119)	>0.9999

## Data Availability

Nucleotide sequences obtained in the present study can be found at Genbank data base (https://www.ncbi.nlm.nih.gov/genbank/, accessed on 29 August 2022) under accession numbers: OP279616, OP279617, OP267564–OP267568.
